# Embryonic Ethanol Exposure Dysregulates BMP and Notch Signaling, Leading to Persistent Atrio-Ventricular Valve Defects in Zebrafish

**DOI:** 10.1371/journal.pone.0161205

**Published:** 2016-08-24

**Authors:** Swapnalee Sarmah, Pooja Muralidharan, James A. Marrs

**Affiliations:** Department of Biology, Indiana University-Purdue University Indianapolis, Indianapolis, IN, 46202, United States of America; Northwestern University, UNITED STATES

## Abstract

Fetal alcohol spectrum disorder (FASD), birth defects associated with ethanol exposure in utero, includes a wide spectrum of congenital heart defects (CHDs), the most prevalent of which are septal and conotruncal defects. Zebrafish FASD model was used to dissect the mechanisms underlying FASD-associated CHDs. Embryonic ethanol exposure (3–24 hours post fertilization) led to defects in atrio-ventricular (AV) valvulogenesis beginning around 37 hpf, a morphogenetic event that arises long after ethanol withdrawal. Valve leaflets of the control embryos comprised two layers of cells confined at the compact atrio-ventricular canal (AVC). Ethanol treated embryos had extended AVC and valve forming cells were found either as rows of cells spanning the AVC or as unorganized clusters near the AV boundary. Ethanol exposure reduced valve precursors at the AVC, but some ventricular cells in ethanol treated embryos exhibited few characteristics of valve precursors. Late staged larvae and juvenile fish exposed to ethanol during embryonic development had faulty AV valves. Examination of AVC morphogenesis regulatory networks revealed that early ethanol exposure disrupted the Bmp signaling gradient in the heart during valve formation. Bmp signaling was prominent at the AVC in controls, but ethanol-exposed embryos displayed active Bmp signaling throughout the ventricle. Ethanol exposure also led to mislocalization of Notch signaling cells in endocardium during AV valve formation. Normally, highly active Notch signaling cells were organized at the AVC. In ethanol-exposed embryos, highly active Notch signaling cells were dispersed throughout the ventricle. At later stages, ethanol-exposed embryos exhibited reduced Wnt/β-catenin activity at the AVC. We conclude that early embryonic ethanol exposure alters Bmp, Notch and other signaling activities during AVC differentiation leading to faulty valve morphogenesis and valve defects persist in juvenile fish.

## Introduction

Fetal alcohol spectrum disorder (FASD) is an inclusive term describing preventable birth defects and disabilities caused by prenatal alcohol exposure. FASD prevalence is as high as 2 to 5 per 100 children, while the more severe fetal alcohol syndrome (FAS) occurs 1 to 2 per 1,000 children [1–3]. In addition to neuro-developmental defects and disabilities, FASD patients exhibit different forms of congenital heart defects (CHDs), including ventricular and/or atrial septal defects, conotruncal defects, and unspecified defects [4]. Most frequently seen CHD in FASD patients are septal defects [4]. CHDs are costly and life threatening global health problems that require on average one invasive surgery, producing considerable morbidity and mortality. Understanding the mechanisms of ethanol-induced heart developmental defects will enable the identification of new therapies or preventive treatments to suppress the deleterious phenotypes.

Cardiogenesis is a complex series of events, involving precise specification and differentiation of cardiac cell lineages, which coordinate cellular morphogenesis events [5, 6]. Cardiac development processes and networks are conserved across vertebrate species [5, 6]. In zebrafish, cardiac progenitors specified around 5 hpf move during gastrulation to form the anterior-lateral plate mesoderm and eventually form the heart tube at 24 hpf. Heart primordia complete a rightward looping to form atrium, ventricle, and atrio-ventricular (AV) canal (AVC). Endocardial progenitors are specified concurrently in a similar embryonic location that migrate alongside cardiac progenitors to form the endocardial lining [5]. AVC differentiation starts around 37 hpf by forming a constriction between the developing atrium and ventricle. Endocardial cells at the AVC undergo differentiation to form endocardial cushions around 48 hpf [5]. At this point, rudimentary valves can prevent retrograde blood flow, albeit inefficiently. Valve function becomes efficient by 72 hpf [7]. Live imaging using high-speed microscopy revealed that two-cell layers thick primitive valve leaflets become apparent between 3 to 4 dpf in zebrafish [7]. Elongation of AV valves occur through 16 days post fertilization (dpf), and maturation is completed in a month by deposition of ECM and thickening of the valves [8]. Adult zebrafish contains four distinct atrio-ventricular valve leaflets oriented anterior, posterior, left and right of the AV orifice [9].

Specific interactions between myocardial and endocardial cells are tightly regulated by gene networks that coordinate critical spatiotemporal morphogenetic mechanisms necessary for functional valve morphogenesis [10, 11]. Combinatorial action of several critical signaling pathways regulate valvulogenesis in zebrafish [6, 11–13]. Detailed analyses revealed that the expression of *bmp4*, which was present in the entire heart tube during early cardiogenesis, becomes restricted to the AVC cardiomyocytes during AVC differentiation [6, 14, 15]. Similarly, *notch1b*, which is expressed in all ventricular endocardial cells, becomes restricted at the AVC endocardium [6, 14, 16]. Hence, the Bmp and Notch activities become prominent at the AVC as compared to the chambers, and drive AVC morphogenetic mechanisms. AV endocardial and myocardial cells produce large amounts of extracellular matrix (ECM) components, the cardiac jelly [10]. Differentiating endocardial cells at the zebrafish AVC change their morphology from squamous to cuboidal, begin expressing cell adhesion molecule Alcama and accumulate high levels of F-actin [14, 17, 18]. Wnt signaling is activated strongly in the developing zebrafish heart valve at 72 hpf [19]. Constitutive activation of Wnt signaling by mutation in the *apc* gene resulted in diffuse expression of AVC markers and massive expansion of endocardial cushions by increased proliferation [19]. Moreover, inhibition of Wnt/β-catenin signaling by overexpression of antagonist *dkk1* inhibited endocardial cushion formation, suggesting precise spatiotemporal role of Wnt/β-catenin signaling in heart valve development [19].

Developmental responses to embryonic ethanol exposure are conserved in different vertebrates [20–26]. In the past three decades, various animal model studies demonstrated that ethanol exposure during development produces heart defects similar to those seen in human [27–31]. Our work illustrated the utility of the zebrafish for studying heart development mechanisms disrupted by ethanol, showing that ethanol exposure during embryogenesis perturbs multiple steps of cardiogenesis, which led to chamber and valve morphogenesis defects [32]. The severity of the defects varies with the stage, duration, and concentration of ethanol exposure. AVC differentiation was especially sensitive to embryonic ethanol exposure [32]. We showed that ethanol exposure during early embryogenesis, long before AVC differentiation led to valvulogenesis defects [32]. Studies using mouse and avian embryos also showed small, defective valves due to bringe-drinking level of ethanol exposure during gastrulation [33, 34]. But it is not known how this early embryonic ethanol exposure causes faulty valve morphogenesis. We hypothesized that ethanol exposure during heart tube morphogenesis (3–24 hour post fertilization; hpf) alters AVC differentiation signaling pathways, leading to valve defects. This ethanol exposure timing was chosen to include gastrulation and somitogenesis periods in zebrafish during which a linear heart tube is formed from specified cardiac progenitors. This period is equivalent to approximately the first 3 weeks of human pregnancy, a period when woman may be unaware of their pregnancy, increasing the risk of prenatal exposure to alcohol. Here, we demonstrate that embryonic ethanol exposure induces valvulogenesis defects leading to faulty valves that persist in juvenile zebrafish. Ethanol exposed embryos fail to restrict the expression of *bmp4* at the AVC causing induction of Bmp activity in the chamber cardiomyocytes and endocardial cells during AVC differentiation. Similarly, endocardial cells failed to restrict Notch activity at the AVC after ethanol exposure, showing widely distributed Notch signaling throughout the ventricular lining. Later, ethanol exposed embryos showed weak Wnt/β-catenin activity at the AVC. We conclude that early embryonic ethanol exposure alters Bmp and Notch activity during AVC differentiation leading to faulty valve morphogenesis and causes valve defects that persist in juvenile fish.

## Methods

### Zebrafish husbandry

Zebrafish [*Danio rerio*; TL and AB strains; *Tg(myl7*:*GFP)*, *Tg(fli1*:*EGFP)*, *Tg(myl7*:*nlsKikGR)* [35], *Tg(bre*:*EGFP)* [36], *Tg(kdrl*:*EGFP)*, *Tg(TP1*:*mCherry)* [37], *Tg(Tcf/Lef-miniP*:*dGFP)* [38] transgenic lines] were raised and housed under standard laboratory conditions [39]. Indiana University-Purdue University Indianapolis School of Science Institutional Animal Care and Use Committee (IACUC) approved animal care and use protocol for this work. *Tg[myl7*:*nlsKikGR]* embryos contains KikGR fluorophore that fluoresces brightly with a green signal. A brief exposure to UV light permanently photoconverts KikGR from green fluorophore to a red fluorophore. *Tg[myl7*:*nlsKikGR]* embryos were exposed to UV light through DAPI filter to convert to red fluorophore.

### Ethanol treatment and raising ethanol treated embryos

Embryos were maintained in embryo medium until 3 hpf. At this time, embryos were transferred to one of the following conditions: embryo medium (control); embryo medium containing 100 mM ethanol (E100) or 150 mM ethanol (E150). At 24 hpf, respective solutions were replaced with fresh embryo medium. Control and ethanol exposed embryos (severely malformed embryos were excluded) were put into the fish system at 5 dpf and raised maintaining same conditions.

### Histology

Histology was performed at Indiana University Histology Core. Embryos were fixed in 4% paraformaldehyde (PFA) in phosphate buffered saline (PBS), dehydrated to 95% ethanol, embedded in JB-4 resin (Polysciences), sectioned at 5 μm thickness using a Leica RM2265 microtome and stained with hematoxylin and eosin or Masson’s trichrome dye.

### Microscopy

Brightfield images on a Leica MZ12 dissecting stereomicroscope were digitally acquired with a color Leica DFC290 camera (Leica Microsystems, Inc., Deerfield IL).

Confocal images were acquired using a Zeiss LSM 700 confocal microscope on an Observer Z1 using 40X 1.1 NA W objective; or 20X 0.8 NA objective. For live imaging, embryos were embedded in 1% low melting agarose containing 0.017% tricane on a coverslip glass bottom microwell dish. Phospho-smad 1,5,9 stained embryos were mounted in low melting agarose and pericardium was manually removed before imaging. 3D renderings were produced from image volumes using Volocity software (PerkinElmer/Invitrogen).

For Selective Plane Illumination Microscopy (SPIM), embryos were embedded in 1% low melting agarose in plastic syringe, and hearts in living embryos were imaged at 100 frames per second (fps) using ImageJ software (NIH software, Bethesda, MD). Movies were made using QuickTime player.

Scanning electron microscopy was performed at Indiana University Electron Microscopy Center. Fish were fixed in 4% PFA, hearts were dissected, post fixed in 2% osmium tetroxide and then followed the procedure as described in Hu et. al. [9]. Atrium was removed and imaged using JOEL 6390 LV microscope.

### Immunofluorescence, F-actin and Wheat Germ Agglutinin staining

Whole-mount immunostaining was performed as previously described with slight modification [40]. For Phospho-smad 1,5,9 antibody staining, embryos were fixed in 4% PFA, washed in PBS, boiled in sodium citrate solution for 2 minute and heated at 60°C for an hour for antigen retrieval. Embryos were blocked and incubated in primary antibody against Alcama (Zn5, Zebrafish International Resource Center) at a dilution of 1:250, Phospho-smad 1,5,9 (Cell Signaling, catalog no. 9511) at a dilution of 1:100, MF20 (Hybridoma bank) at a dilution of 1:50 or β-catenin (Sigma, catalog no. C7207) at a dilution of 1:500. Texas red-conjugated anti-mouse secondary antibody and Alexa Fluor 488 conjugated anti-rabbit secondary antibody was used at 1:100 dilution (Molecular probes/Invitrogen). Texas red or Alexa Fluor 647 conjugated phalloidin (Molecular Probes/Invitrogen) was used 1:100 dilution. Alexa Fluor 555 conjugated Wheat Germ Agglutinin (WGA, Molecular Probes), a lectin that binds to N-acetyl glycosamino glycan, was used at 1μg/ml concentration. TO-PRO-3 (Molecular Probes) was used at 1:1000 dilution for nuclear counterstain. To stain larvae and juvenile fish, hearts were dissected before incubating in the staining solution.

### In situ hybridization

Whole-mount in situ hybridization of zebrafish embryos was performed as described (Sarmah et al., 2010). Digoxigenin-labeled riboprobes for *bmp4* (generously provided by Dr. Ela W. Knapik), *her2* (generously provided by Dr. Pamela Raymond) and *axin2* (Addgene) were synthesized using DIG RNA Labeling Kit (Roche, Indianapolis, IN).

### BODIPY-ceramide staining

Stock solution was made following manufacture’s protocol by dissolving BODIPY-ceramide (D3521; Molecular probe) in DMSO. BODIPY-ceramide staining solution was made by diluting the stock solution to 0.1 mM by adding embryo medium containing 1% DMSO. Embryos were dechorionated manually and incubated in staining solution from 30 to 48 hpf (15 embryos / 1 ml solution). Embryos were washed in embryo medium before imaging.

### Statistical Analysis

Statistical analyses comparing control and ethanol treated groups were performed using unpaired two-tailed Student’s t-test (GraphPad Software, San Diego, CA).

## Results

### Early embryonic ethanol exposure leads to defects in atrio-ventricular valvulogenesis, a morphogenetic process that takes place long after ethanol withdrawal

**Fig 1:**
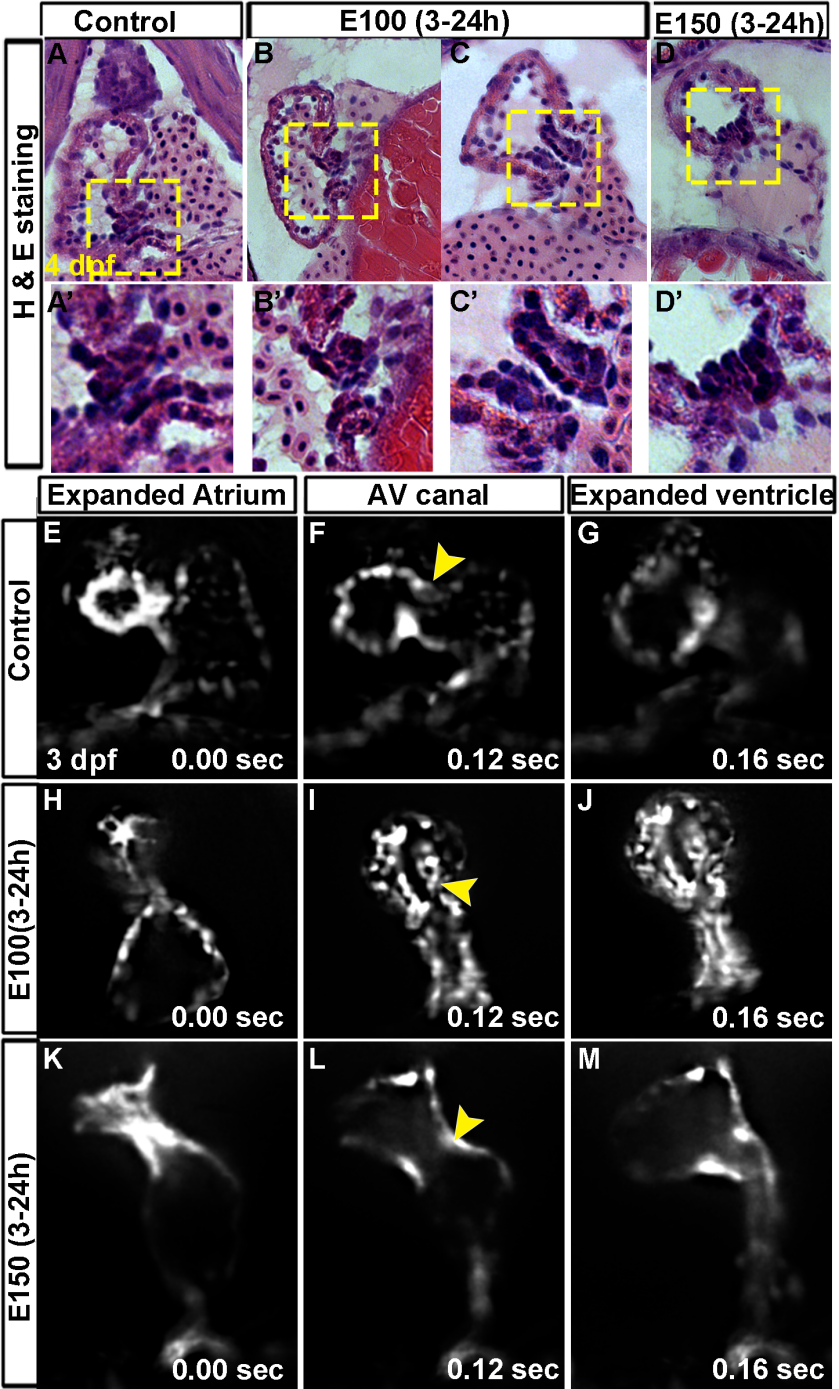
Embryonic ethanol exposure led to defective AV valve formation in zebrafish. (A-D') Hematoxylin and eosin stained coronal sections of 4 dpf zebrafish hearts showed two layers of valve forming cells confined at the AVC in control embryos (A, A'); unorganized clusters of cells at the AV boundary in E100 ethanol treated embryos (B, B'); rows of cells at the extended AVC in E100 ethanol treated embryos (C, C'); and unorganized clusters of cells at the AV boundary in E150 ethanol treated embryos (D, D'). High magnification images of the boxed areas (A'-D'). (E-M) SPIM imaging of beating hearts of *Tg(fli1*:*EGFP)* embryos showed completely filled atrium (E, H, K) and ventricular (G, J, M) chambers before ejection of blood in control (E, G) and ethanol treated embryos (H, J, K, M). (F) Control beating heart at the relaxed state showed valve leaflets at the superior and inferior aspects of AVC (F; arrowheads). Ethanol treated embryos showed long valve forming cells extending into the ventricle chamber (E100 ethanol, I) or no valve forming cells at the AVC in E150 treated embryos (L).

To understand the effects of early ethanol exposure on valve morphogenesis, zebrafish embryos were exposed to ethanol (E100 or E150) from 3 to 24 hpf, and then embryos were returned to normal embryo medium. Hearts and AV valves were analyzed at later stages. All ethanol treated embryos developed pericardial edema, showed reduced cardiac looping at 2 dpf (data not shown), as shown in our previous studies [25, 32]. Higher concentration of ethanol treatment occasionally produced severely defective embryos with a straight heart. To examine the effect of ethanol exposure (3–24 hpf) on AV valve morphogenesis, 4 dpf control and ethanol exposed embryos were sectioned coronally and stained with hematoxylin and eosin. Valve leaflets of the control embryos (6/6 embryos) comprised two layers of cells those were confined at the AVC. Ethanol treated embryos had extended AVC. Those embryos showed either unorganized clusters of cells near the AV boundary (5/12) or rows of cells at the extended AVC (7/12) (Fig 1A and 1B). Severely defective embryos with straight heart showed fewer unorganized cells at the AVC (Fig 1D).

To better understand the morphology of the developing valve leaflets in live embryos, 3 dpf *Tg(fli1*:*EGFP)* embryos, labeling endothelium and endocardium, were examined using high-speed SPIM. SPIM images showed completely filled chambers and the AVC at the open and closed states (Fig 1E–1M). Control beating heart had small valve leaflets at the superior and inferior aspects of AVC (Fig 1F, and S1 Movie). Ethanol treated embryos showed long valve leaflets extending into the ventricle chamber which occupied most of the ventricular volume as seen right before ventricular ejection (Fig 1H–1J, and S2 Movie). Embryos with straight heart (severely defective) pumped blood by peristalsis movement of the heart (Fig 1K–1M, and S3 Movie). Those embryos did not show obvious valve leaflets at the AVC. Control and ethanol treated embryos analyzed using SPIM also showed that cardiac cycles of the embryos were completed in similar time frames (Control, 0.25 sec; E100, 0.25 sec; E150, 0.26 sec; based on 2 embryos per group). To further analyze ethanol effect on cardiac function, heartbeat of control and ethanol treated embryos were measured at 36 and 48 hpf. At 36 hpf, heartbeats of E100 embryos were not statistically significantly different from control, however, E150 embryos showed significantly increased heartbeat: mean heartbeats- Control = 128.9±16.88, E100 = 133.36±7.46, E150 = 142.9±1.7; number of embryos = 14/group; P value: Control vs. E100, 0.4329; Control vs. E150, 0.018 (results from two individual experiments). Measurement at 48 hpf also showed no statistically significant difference in the heartbeat between control and E100 groups, but heartbeat of E150 group was significantly more than the control group: Control = 139.29±7.75, E100 = 137.38±9.17, E150 = 145.63±8.01; number of embryos = 16/group; P value: Control vs. E100, 0.5459; Control vs. E150, 0.0366 (results from two individual experiments). Taken together, these data indicate that early ethanol exposure severely interrupts AV valve morphogenesis and exposure to high dose of ethanol alters cardiac function in embryos.

### Ethanol-exposure reduced precursors of valves at the AVC and produced ectopic valve-like cells in the ventricular endocardial lining

**Fig 2:**
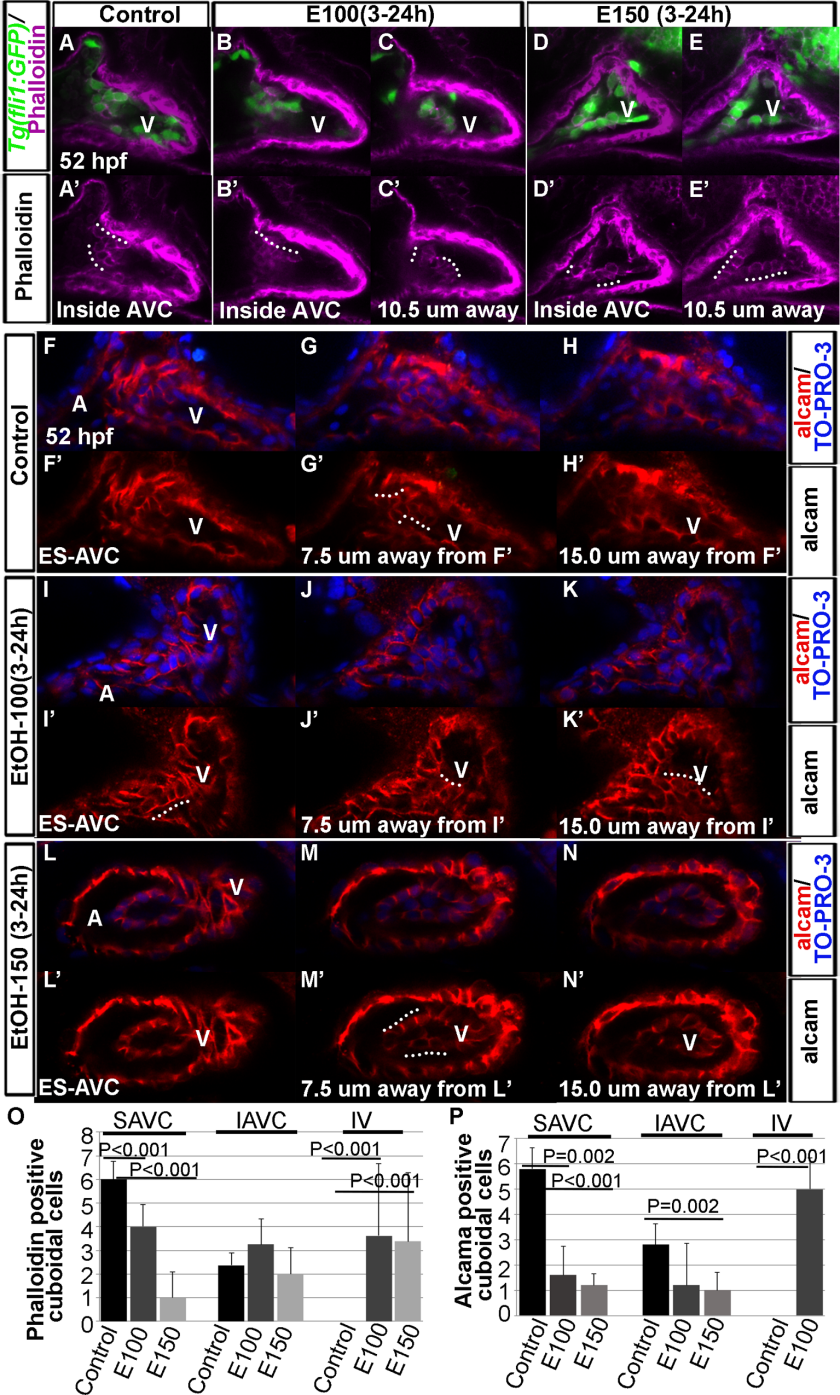
Ethanol-exposure reduced precursors of valves at the AVC and produced ectopic valve-like cells in the ventricular endocardial lining. (A-E’) Confocal sections of phalloidin stained *Tg(fli1*:*EGFP)* embryos showed differentiated cuboidal valve precursors inside AVC in control embryos (A, A’); E100 ethanol treated embryos showed phalloidin stained cuboidal cells inside AVC (B, B’) and inside the ventricle (10.5 μM away from the section B; C, C’); E150 ethanol treated embryos showed fewer phalloidin stained cuboidal cells at the AVC (D, D’) but more cells inside the ventricle (10.5 μM away from the section D; E, E’). White dotted lines show the rows of cuboidal cells. (F-N’) Confocal sections of anti-Alcama antibody stained embryos showing Alcama expression in the endocardial cells at the surface of the endocardium at AVC (F, F’, I, I’, L, L’), 7.5 μM (G, G’, J, J’, M, M’) and 15.0 μM away from surface endocardial cell sections (H, H’, K, K’, N, N’) in control and ethanol-exposed embryos. Control embryos showed Alcama positive cuboidal cells at 7.5 μM away from the surface AVC endocardium (G, G’); ethanol-exposed embryos showed Alcama positive cuboidal cells at the surface of AVC endocardium (I, I’) as well as 7.5 μM and 15 μM away from the AVC (J-K’, M-M’). White dotted lines show rows of cuboidal cells. A: Atrium, V: Ventricle, ES-AVC: Endocardial cells at the surface of AVC. (O, P) Graphs depict the number of phalloidin positive (O) and Alcama positive (P) cuboidal cells at the superior (SAVC) and inferior (IAVC) aspects of AVC, and inside the ventricle (IV) in control and ethanol-treated embryos. Statistically significant P values are shown in the graphs.

Zebrafish endocardial cells at the AVC express Alcama and accumulate high levels of F-actin upon AVC differentiation [14]. At 55 hpf, a single sheet of differentiated endocardial cells expressing both F-actin and Alcama become cuboidal in shape, which are the precursors of the heart valves [6, 14]. To understand the AVC differentiation in ethanol exposed embryos, F-actin and Alcama expression were examined in *Tg(fli1*:*EGFP)* embryos at 52 hpf. In control embryos, the endocardial cells at the AVC showed strong phalloidin staining (F-actin). Moreover, a row consisting 5–7 cells at the superior aspect and another consisting 3–4 cells (based on the cell count in 8 embryos) at the inferior aspect of AVC became cuboidal in shape (Fig 2A and 2A’). Ventricular chamber endocardial cells did not exhibit phalloidin staining. Ethanol treated embryos showed mislocalization of these cells. Phalloidin-stained endocardial cells were found inside the ventricular chamber in addition to AVC (Fig 2B–2E’). However, fewer phalloidin positive AVC endocardial cells were cuboidal in shape in ethanol treated embryos. (E100 treated embryos: superior aspect of AVC, 3–5 cells; inferior aspect of AVC, 1–4 cells; and E150 treated embryos: superior aspect of AVC, 0–2 cells; inferior aspect of AVC, 2–3 cells; based on cell count in 8 embryos of each group). Occasionally cuboidal cells were disorganized and did not arrange in a single sheet of cells. Although not all, some of the phalloidin positive endocardial cells found inside the ventricular chamber were also cuboidal in shape (E100 treated embryos: inside the ventricle, 4–8 cells, and E150 treated embryos: inside the ventricle, 2–7; based on cell count in 8 embryos of each group) (Fig 2B–2E’). Average phalloidin positive cuboidal cells in superior and inferior aspects of AVC and inside the ventricle of control and ethanol treated embryos are shown in the graph (Fig 2O).

The anti-Alcama antibody staining showed similar results. Optical coronal sections of the heart of control embryos showed Alcama positive endocardial cells at the surface of AVC as well as inside the AV canal, but Alcama was not detected in the chamber endocardium (Fig 2F–2H’). In control embryos, there were 5–7 cuboidal Alcama positive endocardial cells at the superior aspects of AVC and 2–3 cuboidal Alcama positive endocardial cells at the inferior aspects of AVC (Fig 2G and 2G’). Ethanol exposure changed the Alcama expression pattern in the heart showing Alcama positive cells in the ventricle in addition to AVC (Fig 2I–2N’). Also, the number of cuboidal Alcama positive cells at the AVC was reduced (E100 treated embryos: superior aspect of AVC, 1–3 cells; inferior aspect of AVC, 0–4 cells, and E150 treated embryos: superior aspect of AVC, 0–2 cells; inferior aspect of AVC, 0–2 cells). Alcama positive cuboidal cells were also seen in the ventricular endocardium (Fig 2P). Average Alcama positive cuboidal cells at superior and inferior aspects of AVC of control and ethanol treated embryos are depicted in the graph (Fig 2P). Together, these findings show that ethanol exposure reduces the number of valve precursors at the AVC, but induces ectopic cells in the ventricle that adopt AVC cell characters. However, they are not identical to the normal differentiated AVC endocardial cells.

### Ethanol induced valvulogenesis defects led to faulty valves in juvenile zebrafish

**Fig 3:**
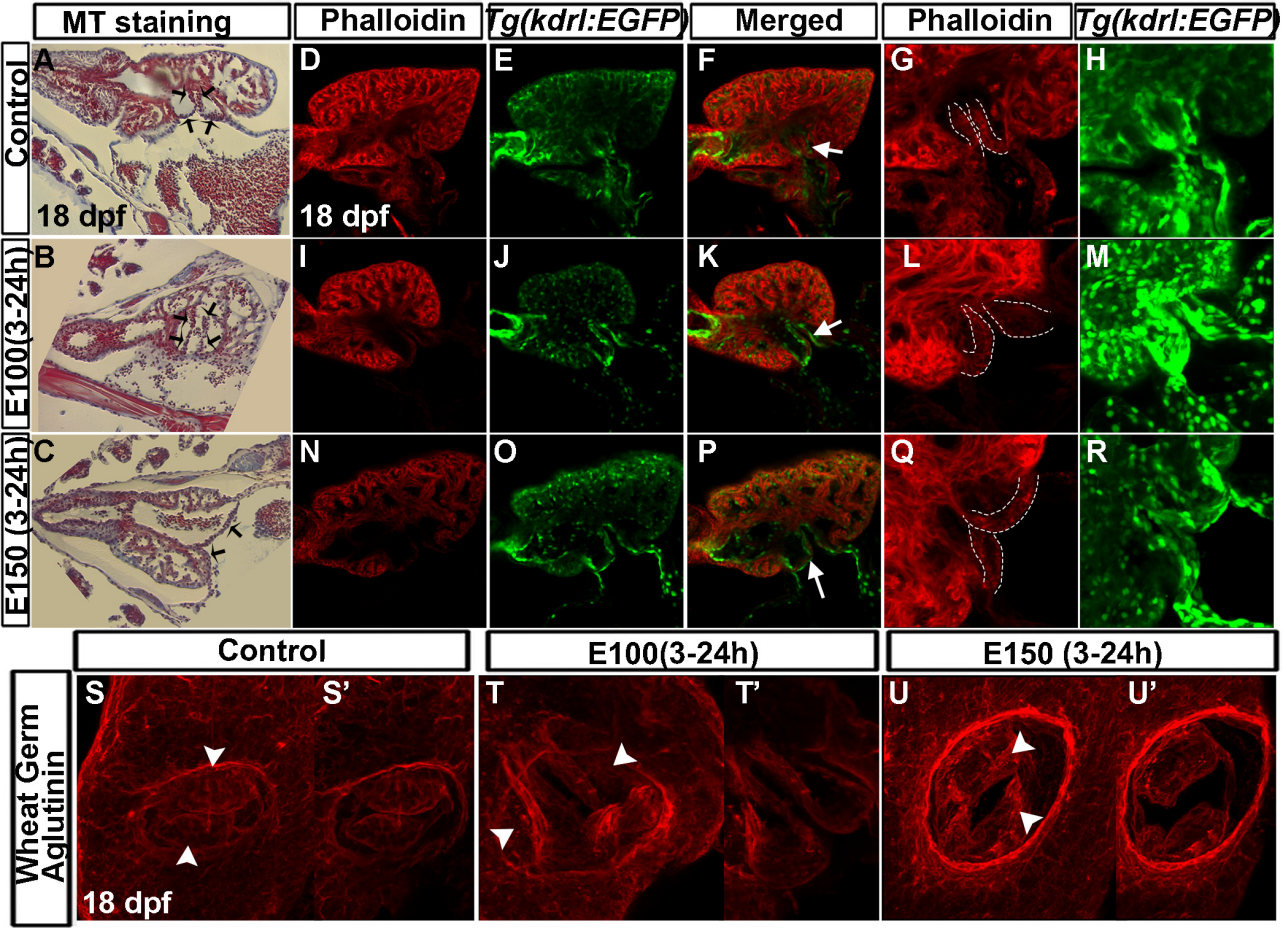
Late-staged zebrafish larvae exposed to ethanol during embryogenesis exhibited defective valve leaflets. (A-C) Masson's trichrome stained coronal heart sections of 18 dpf larvae showed short and curved valve leaflets in control larvae (A), and longer and straight valve leaflets in larvae exposed to ethanol during embryogenesis (B, C). Black arrows: valve leaflets. (D-R) Single optical sagittal section of the heart of phalloidin stained *Tg(kdrl*:*EGFP)* larvae showed short AV valve leaflets in control larvae (D-F) and long valve leaflets in ethanol exposed larvae (I-K, N-P). White arrows: valve leaflets. 3D-rendering of the confocal sections showed narrow valve leaflets in the control (G, H) and thickened valve leaflets in ethanol exposed larvae (L, M, Q, R). White dotted lines demarcate valve leaflets. (S-U’) 3D-rendering of the optical sections at the AV junction of WGA labeled larvae showed two valve cusps in control larvae (S, S’) and deformed valve cusps in ethanol-exposed larvae (T-U’); S (54 μM thick), T (79.38 μM thick), U: 3D (81.9 μM thick) show the rendering of total number of Z sections. S’, T’, U’: 3D-rendering of same number of Z sections (25.2 μM thick). MS: Masson’s trichrome, Arrowhead: valve cusps.

It is crucial to determine whether ethanol-induced defects persist in much later stages. For that, we undertook a longitudinal study to examine valve morphology at different stages of the fish life. Control and ethanol exposed larvae were raised. Although not all ethanol exposed embryos survived, a high number of embryos reached juvenile stage (around 70% of E100 and 50% of E150 ethanol exposed embryos). Ethanol-exposed larvae reaching 18 dpf stage showed significant defects in the AV valve morphology. Masson's trichrome stained serial coronal heart sections of 18 dpf larvae showed that the developing valve leaflets were curved and short in control larvae, but were longer and often straight in ethanol exposed larvae (Fig 3A–3C).

Phalloidin staining was performed on endothelium and endocardium marked *Tg(kdrl*:*EGFP)* larvae and imaged using confocal microscopy. Optical sagittal sections through the heart showed two short and narrow AV valve leaflets in 18 dpf control larvae. However, ethanol exposed larvae showed long and thickened valve leaflets (Fig 3D–3Q). To dissect the defects in more detail, *Tg(kdrl*:*EGFP)* larvae were stained with WGA (the lectin binds to n-acetyl glycosamino glycan). Atrium was removed manually before confocal imaging and AV valves were imaged. Comparison of 3D rendering of confocal sections of control and ethanol treated larvae showed that the AV valves of ethanol treated larvae were severely malformed and AV junctions were much wider than the control (Fig 3S–3U’). Moreover, the WGA labeling in the AV junction was stronger in ethanol exposed larvae compared to control suggesting the composition of glycoproteins in cells of AV junction was different.

**Fig 4:**
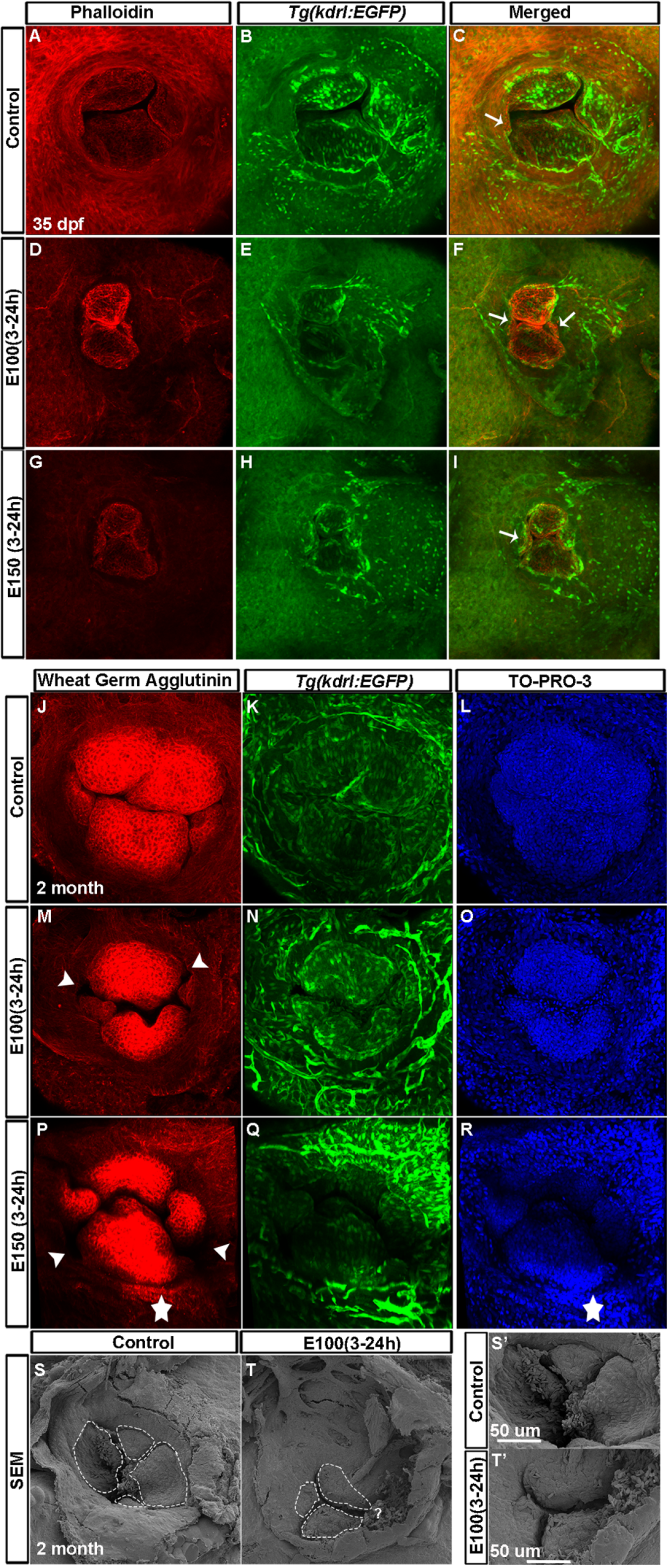
Juvenile fish exposed to ethanol during embryonic development exhibited defective valves. (A-I) Phalloidin stained *Tg(kdrl*:*EGFP)* fish showed two bigger valve cusps at the superior and inferior position, and two smaller leaflets oriented left and right of the AV orifice in control fish at 35 dpf (A-C); ethanol-exposed fish showed four smaller and irregular-shaped valve cusps (D-I). Arrows: smaller cusps. (J-R) WGA stained 2 months old *Tg(kdrl*:*EGFP)* fish exposed to ethanol during embryonic development showed four well-organized nicely-shaped valve cusps in control fish (J-L) and smaller, malformed valves in ethanol exposed fish (M-R). Arrowheads point to the holes between valve cusps; star demarcates the region strongly labeled by WGA at the AV orifice. (S-T’) Scanning electron microscopy of two months old fish showed four valve cusps in control fish (S-S’) and three smaller valve leaflets in ethanol treated fish (T, T’). S’ and T’: high magnification views of S and T; “?” point to the region of the possible fourth cusp.

Juvenile fish AV valves were then analyzed using phalloidin staining in *Tg(kdrl*:*EGFP)* fish at 35 dpf. Since the maturation of valves depends on the larval size [8], we examined control and ethanol treated fish with similar weights. Control fish having similar weight (0.035±0.0047 g) showed four valve cusps, which include two larger valve leaflets oriented anterior and posterior (superior and inferior), and two smaller leaflets oriented left and right of the AV orifice (Fig 4A–4C). Ethanol exposed fish of similar weight (0.042±0.011 g) also showed four valve leaflets, but strikingly, the leaflets were smaller than the control and the shape of the leaflets were different, producing irregular correspondence between adjacent leaflets (Fig 4D–4I).

Next, we examined AV valves in 2 month old *Tg(kdrl*:*EGFP)* fish exposed to ethanol during embryonic development. WGA stained fish showed a similar result that observed in one-month old fish. Valve leaflets in ethanol exposed fish were small and the shape of the adjacent leaflets do not closely correspond to allow complete closure of the AV junction (Fig 4J–4R). Scanning electron microscopy of two-month old fish also showed smaller valve leaflets in ethanol treated fish, confirming the confocal microscopy result (Fig 4S–4T’). Taken together, embryonic ethanol exposure leads to valve morphogenesis defects producing faulty AV valve that persist to juvenile stages.

### Ethanol mislocalized Bmp activity that was not restricted to the AVC in embryonic hearts

**Fig 5:**
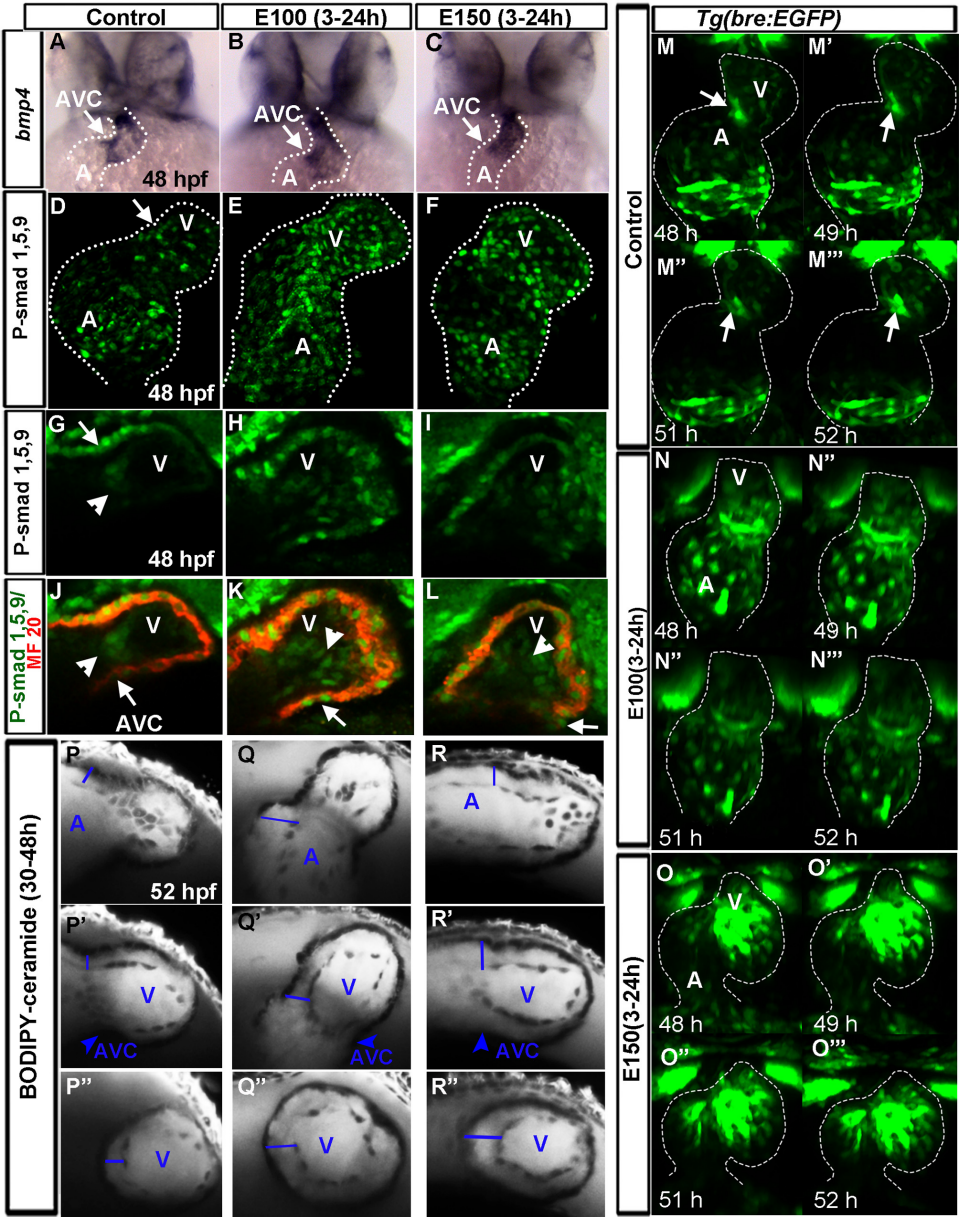
Ethanol exposure led to the failure of suppression of Bmp activity from the chamber cells during valvulogenesis. (A-C) In situ hybridization detecting *bmp4* expression showed restricted expression at the AV boundary and outflow tract in control embryo (A) and widely distributed throughout the ventricle in ethanol treated embryos (B, C). (D-F) 3D reconstruction of confocal sections of phospho-Smad-1/5/9 immunostained embryos showed Bmp responsive phospho-Smad-1/5/9 positive cardiomyocytes at the base of the atrium, near AVC and inner curvature of the ventricle (white arrow) in the control embryo (D); phospho-Smad-1/5/9 positive cardiomyocytes were seen throughout the venricle in ethanol exposed embryos. *Note: Please see S1 Fig for high magnification images.* (G-L) Single Z-sections of phospho Smad-1/5/9 and MF20 (cardiac myosin) double immunostained embryos at the AV boundary showed phospho-Smad-1/5/9 positive endocardial cells at AVC in the control embryos (G, J) and throughout the entire ventricular endocardium in ethanol exposed embryos (H, I, K, L). Arrowheads: phospho-Smad-1/5/9 positive endocardial cells, Arrows: AVC myocardium. Note phospho-Smad-1/5/9 positive myocardial cells at the AVC towards the inner curvature of the ventricle in control embryo (G; arrow) and all ventricular myocardial cells in ethanol-exposed embryos (H, I). (M-O”) Time lapse images of *Tg(bre*:*EGFP)* embryos from 48–52 hpf showed progressive loss of GFP signal from chamber cardiomyocytes, and strong GFP signal at the AV canal towards the inner curvature (arrow) and at the base of the atrium in control embryos (M-M’”); GFP signal was less suppressed from the chamber cardiomyocytes in E100 ethanol exposed embryos (N-N’”) and not suppressed in E150 ethanol exposed embryos (O-O’”). (P-R”) BODIPY-ceramide labeled embryos showed expansion of cardiac jelly (blue line) region between myocardium and endocardium in the atrium (P-R), AVC (P’-R’) and in the ventricle (P”-R”) in ethanol-exposed embryos (Q-R”) compared to control (P-P”). A: atrium, V: ventricle. White dotted line demarcates the heart.

To examine the mechanisms of the ethanol induced AV valve defects, AVC morphogenesis regulatory networks were examined in zebrafish embryos, based on previous studies [6, 10, 11]. Bmp plays diverse roles in valve development [6]. Zebrafish heart primordia begin to express Bmp ligand *bmp4*, which remains present throughout the anteroposterior extent of the heart during heart tube morphogenesis. Later, *bmp4* expression becomes restricted at the AVC during its differentiation (around 37 hpf). Analysis of *bmp4* at 24 hpf showed its expression in the linear heart tube in the control embryos. Ethanol exposed embryos showed relatively uniform *bmp4* expression in the forming heart tube at this stage (data not shown). At 48 hpf, *bmp4* expression was restricted at the AV boundary and near the outflow tract (OFT) in control embryos (Fig 5A). However, ethanol exposed embryos did not show restricted expression of *bmp4* at the AVC. The expression of *bmp4* was evident in the ventricular cardiomyocytes in addition to AVC in ethanol exposed embryos (Fig 5B and 5C).

To examine Bmp signaling in the ethanol exposed embryos during AVC differentiation, phospho-Smad-1/5/9 (activated Bmp/Smad transcription factor activity) and MF20 (monoclonal against cardiac myosin heavy chain) double label immunostaining was performed at 48 hpf. Three-dimensional reconstruction of confocal z-sections showed more phospho-Smad-1/5/9-labeled cardiomyocytes at the base of the atrium, at the AVC and inner curvature of the ventricle in the control embryos (Fig 5D, please see S1A–S1C Fig, S1A’ Fig for high magnification images). Ethanol exposed embryos did not show regionalization of phospho-Smad-1/5/9 staining during AVC differentiation. These cardiomyocytes with activated Bmp/Smad transcription factor activity, were evident throughout the heart of ethanol exposed embryos (Fig 5E and 5F, S1D–S1I Fig, S1D’ Fig, S1G’ Fig). Optical sections near AV boundary showed fewer phospho-Smad-1/5/9 labeled endocardial cells at the AVC in the control embryos. In contrast, phospho-Smad-1/5/9 labeled endocardial cells were seen throughout the entire ventricular endocardium in ethanol exposed embryos (Fig 5G–5L). Next, we examined the Bmp signaling in the heart in live embryos using transgenic line *Tg(bre*:*EGFP)*, which express GFP in response to a Bmp response element that reports Bmp/Smad signaling [36]. Time course analyses of *Tg(bre*:*EGFP)* embryos from 48 hpf to 52 hpf showed that GFP signal was progressively lost from chamber cardiomyocytes but the GFP positive cells were seen in AV canal, in the inner curvature of the ventricle and at the base of the atrium in the control embryos (Fig 5M–5M”‘). However, the GFP signal was either not suppressed or was less suppressed from the chamber cardiomyocytes in ethanol exposed embryos during this period (Fig 5N–5O”‘).

A previous study showed that failure to repress *bmp4* expression from the chambers during AVC differentiation caused increased expression of *has2* (hyaluronic acid synthetase 2) that subsequently led to the expansion of cardiac jelly between myocardium and endocardium [17]. To analyze the cardiac jelly region after ethanol exposure, embryos were incubated in BODIPY-ceramide solution from 30–48 hpf and whole heart was imaged using confocal microscope. BODIPY-ceramide labels blood serum, cardiac jelly and cell membrane [41, 42]. Ethanol exposed embryos showed an expansion of cardiac jelly region between myocardium and endocardium in the atrium, AVC and ventricle (Fig 5P–5R”).

**Fig 6:**
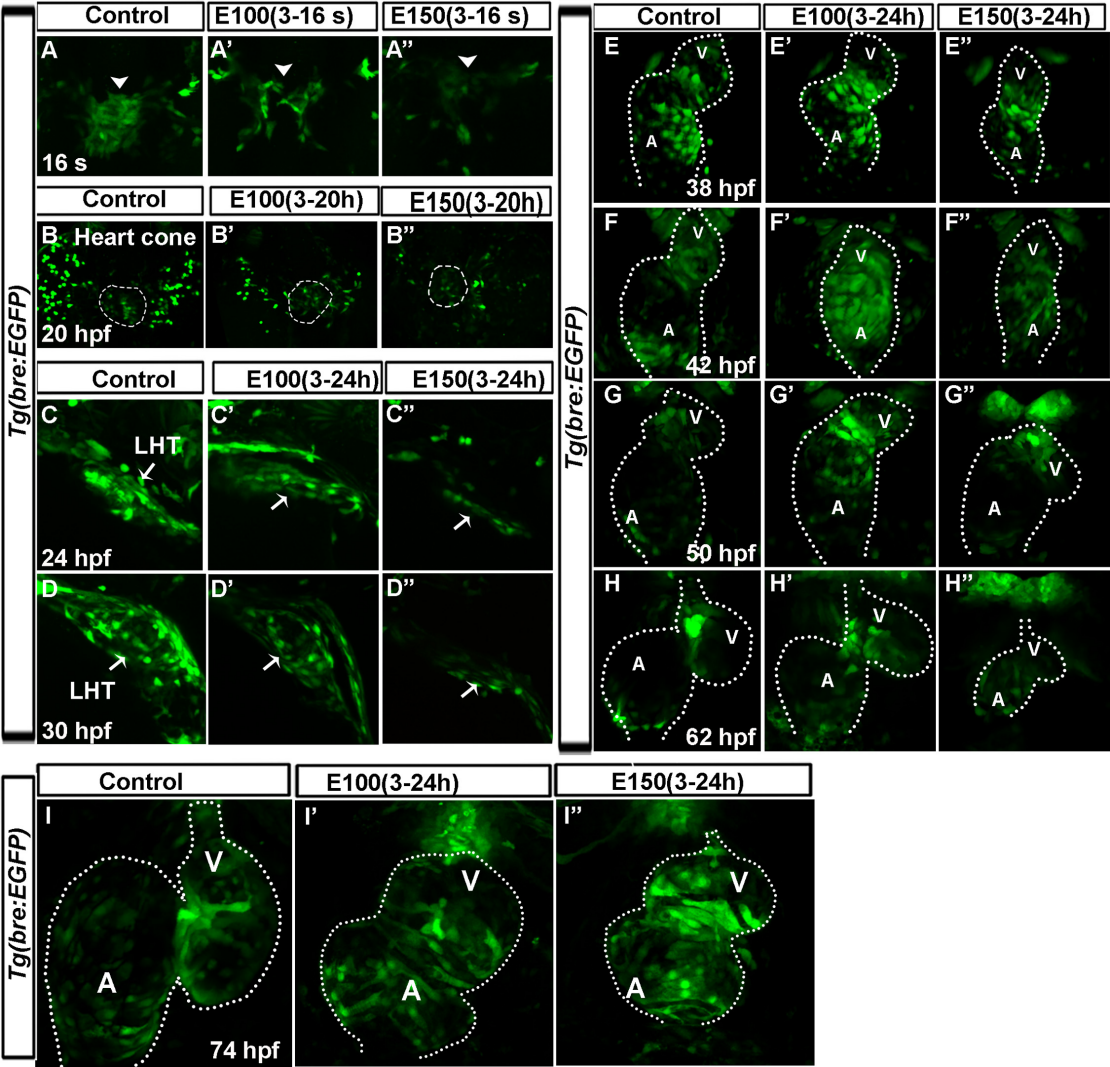
Ethanol exposure reduced Bmp activity during heart tube morphogenesis, and later during valvulogenesis, those embryos lost regionalization of Bmp activity at the AVC. (A-B”) *Tg(bre*:*EGFP)* embryos showed reduced GFP label in the cardiac primordia in ethanol exposed embryos (A’-B”) relative to control (A, B) at 16 somite (16s; 17 hpf; A-A”) and heart cone stage (20 hpf; B-B”). Arrowheads: cardiomyocytes; dotted line demarcates the heart cone. (C-D”) Reduced GFP signaling was observed in the linear heart tube (LHT) of ethanol exposed embryos at 24 (C’, C”) and 30 hpf (D’, D”) compared to control (C, D). (E-I”) Examination of Bmp activity from 38–74 hpf showed progressively restricted Bmp activity in the chamber cardiomyocytes and strong activity at the AVC in control embryos (E-I); ethanol treated embryos showed lack of restriction of Bmp activity at the AVC (E’-I”) and occasional weak BMP activity in the heart (H”). Dotted line demarcates the heart. A: Atrium, V: Ventricle.

Since Bmp plays crucial roles throughout cardiogenesis including AV cushion development, ethanol effects on Bmp signaling were examined from 17 hpf until 75 hpf, a period encompassing the formation of heart cone until two-chambered heart. Reduced Bmp signaling was observed in the heart cone and heart tube of ethanol exposed embryos at 17–30 hpf compared to control (Fig 6A–6D”). Around 38 hpf, control embryos started to restrict Bmp activity from chamber cardiomyocytes (Fig 6E), which was progressively lost from the chamber cardiomyocytes (Fig 6E–6H). However, the GFP signal was either not suppressed or was less suppressed from the chamber cardiomyocytes in ethanol exposed embryos during this period (Fig 6E’–6H”). Examination of the embryos at later developmental stages showed that chamber cardiomyocytes remain GFP positive even at 3 dpf in ethanol exposed embryos (Fig 6I–6I”). Although, most ethanol treated embryonic hearts showed GFP intensity that was comparable to control, severely defective embryos showed relatively weaker GFP intensity from a Bmp activity responsive promoter (Fig 6H”). These findings indicated that ethanol exposure dysregulates Bmp activity during heart morphogenesis. Ethanol induced Bmp signaling defects are two-fold: initially, during heart tube morphogenesis, ethanol exposure reduces Bmp activity; and later, during AVC patterning and differentiation, ethanol exposure leads to failure of suppression of Bmp activity from the chamber cardiomyocytes.

### Ectopic Notch activity was evident in the ventricular endocardium in ethanol exposed embryos

**Fig 7:**
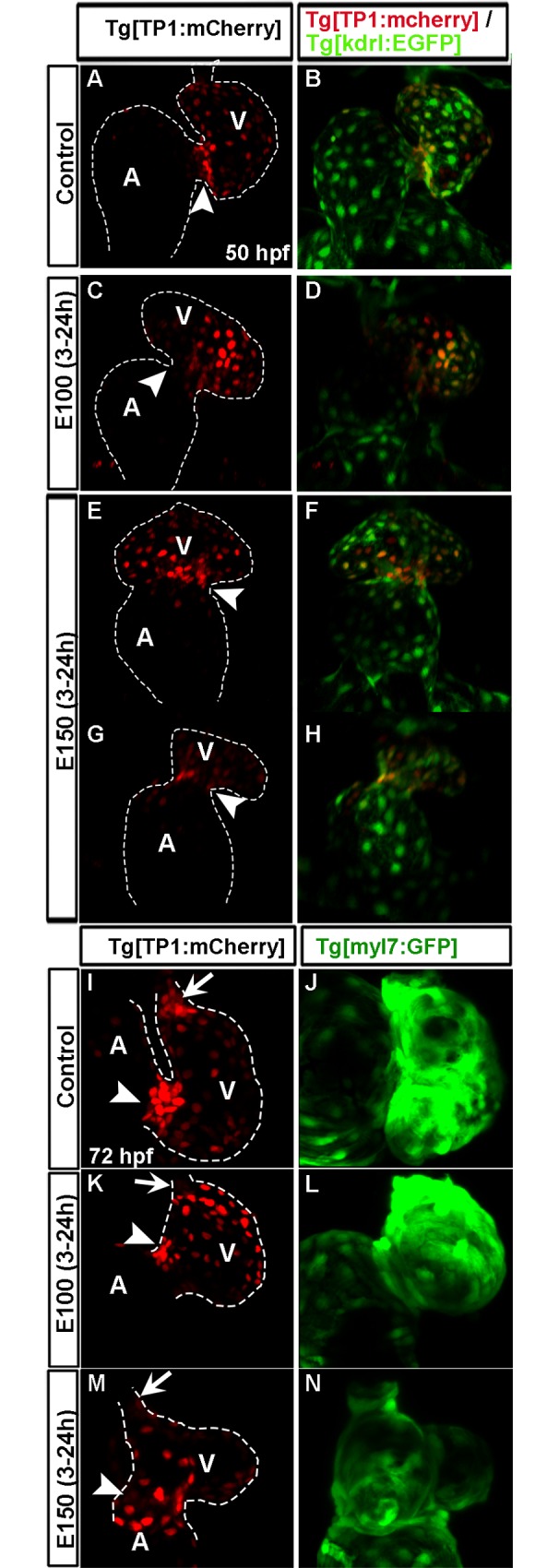
High Notch activity was not restricted at the AVC endocardial cells in ethanol exposed embryos. (A-H) *Tg(TP1*:*mCherry);Tg(kdrl*:*EGFP)* double transgenic embryos showed compact cluster of strong mCherry labeled cells at the AVC and weak mCherry labeled cells in the ventricular endocardial lining in control embryo at 50 hpf (A, B); ethanol treated embryos had a mix of strong and weak mCherry labeled cells in the endocardium and no cluster of strong mCherry labeled cell at the AVC (C-H); severely defective embryo exhibited weaker mCherry labeling relative to the control (G, H). (I-N) *Tg(TP1*:*mCherry);Tg(myl7*:*GFP)* double transgenic embryos showed compact clusters of strong mCherry labeled cells at the AVC and OFT at 72 hpf in control (I) and no clustering in ethanol treated embryos (K, M); green fluorescence in *Tg(TP1*:*mCherry);Tg(myl7*:*GFP)* embryos showed myocardium at 72 hpf (J, L, N). A: Atrium, V: Ventricle, Arrowheads: AVC, Arrows: OFT.

Notch signaling was implicated to play multiple roles in zebrafish AVC development including restriction of AVC differentiation in the ventricle. Similar to the prominent Bmp activity at the AVC during differentiation, Notch signaling activity becomes restricted at the AVC endocardial cells. Altering Notch activity during cardiogenesis leads to valvulogenesis defects [6, 14]. Notch activity during AVC formation was examined in *Tg(TP1*:*mCherry);Tg(kdrl*:*EGFP)* and *Tg(TP1*:*mCherry);Tg(myl7*:*GFP)* double transgenic embryos. *Tg(TP1*:*mCherry)* transgene labels Notch active cells and *Tg(myl7*:*GFP)* labels cardiomyocytes. Live imaging of *Tg(TP1*:*mCherry);Tg(kdrl*:*EGFP)* control embryos at 50 hpf showed that mCherry labeled Notch active cells present in the ventricular endocardium can be grouped into two categories: (i) compact clusters of strongly labeled mCherry positive cells (high Notch activity) present at the AVC, and (ii) weakly labeled mCherry positive cells (low Notch activity) present in the ventricular endocardial lining (Fig 7A and 7B). The pattern of the localization of high and low Notch active cells in heart during this period was lost in ethanol exposed embryos. Both high and low Notch active cells were found in the endocardium irrespective of the location, and there was no cluster of high Notch-active cells at the AVC (Fig 7C–7H). Severely defective embryos exhibited weaker mCherry labeled cells, suggesting low notch-activity than the control (Fig 7G and 7H). At 72 hpf, control *Tg(TP1*:*mCherry);Tg(myl7*:*GFP)* embryos showed more prominent clustering of high Notch-active cells at the AVC and OFT. In contrast, ethanol exposed embryos showed persistent defects in Notch-active cell distribution at later stages (Fig 7I–7N). The expression of Notch target *her2* was examined at 48 hpf. Control embryos showed a faint expression of *her2* in the heart, but ethanol treated embryos showed *her2* expression throughout the ventricle (S2A–S2C Fig).

**Fig 8:**
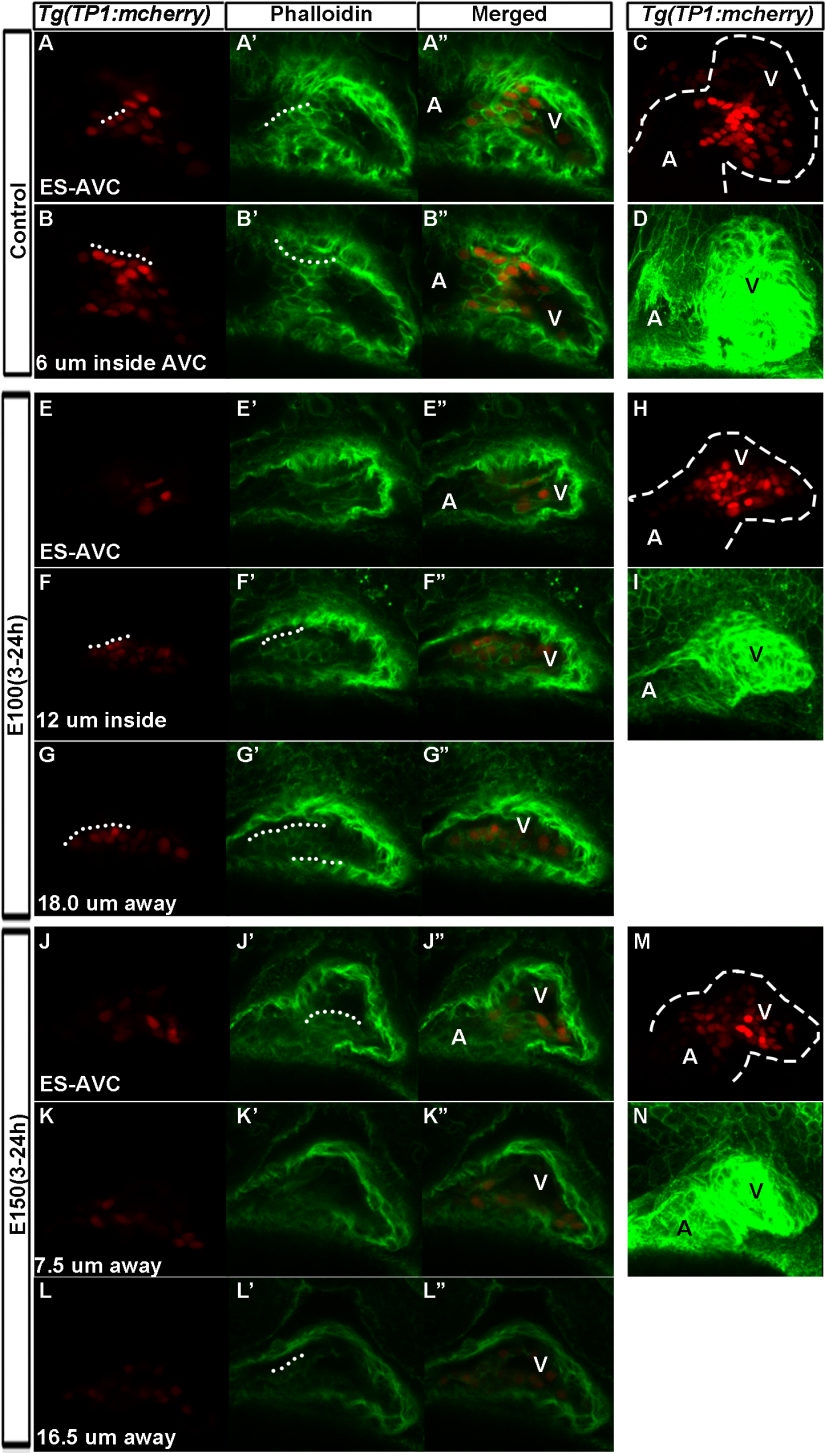
High Notch-active cells present in the ventricular endocardium in ethanol exposed embryos showed F-actin expression. (A-B”) Texas-red phalloidin stained 52 hpf *Tg(TP1*:*mCherry)* embryos showed strong mCherry labelled endocardial cells confined at the AVC in control embryo (A-A”: at the surface of AVC; (B-B”): cells inside AVC); Strong Notch positive cells along with a few neighboring cells expressed high level of F-actin and were cuboidal in shape (White dotted lines). (C) 3D rendering of optical Z-sections through heart showing distribution of strong and weak Notch active cells in the heart of control embryo. (D) 3D rendering of phalloidin stained embryos showing shape of the control embryo heart. (E-G”, J-L”) Ethanol treated and Texas-red phalloidin stained *Tg(TP1*:*mCherry)* embryos showed strong mCherry labelled endocardial cells at a lesser extent at the AVC endocardium (E-E”, J-J”), but more inside the ventricle (F-G”, K-L”). Notch active cells in the chamber endocardium showed ectopic F-actin labeling (F’-G”, K’-L”). White dotted lines show cuboidal cells. (H, M) 3D rendering of optical Z-sections through heart showing distribution of strong and weak Notch active cells in the heart of ethanol treated embryos. (I, N) 3D rendering phalloidin stained embryos showing the shape of the heart of ethanol treated embryos compared to control (D). ES-AVC: Endocardial cells at the surface of AVC, A: Atrium, V: Ventricle.

Notch signaling activates epithelial-to-mesenchymal transition, which is needed to produce valve primordia [43].To examine whether highly Notch active cells present in the ventricle of ethanol exposed embryos lead to ectopic expression of F-actin in the ventricular endocardium, Texas-red phalloidin staining was performed in *Tg(TP1*:*mCherry)* embryos. Confocal sectioning of the stained embryos showed high Notch active cells in the endocardium at the AVC express high level of F-actin. Inside the AVC of control embryos, strongly labelled mCherry positive cells lined up in a row, and those highly Notch active cells along with a few neighboring cells expressed high level of F-actin and were cuboidal in shape (Fig 8A’–8B”). Strong mCherry positive cells were found at a lesser extent in the endocardium at the surface of AVC and inside the AVC of E100 ethanol treated embryos, and rarely detected in E150 ethanol treated embryos (Fig 8E–8F” and 8J–8K”). Accordingly, there were fewer cuboidal F-actin positive cells residing at the AVC of ethanol treated embryos (Fig 8F’ and 8K’). Moreover, cells with high Notch active cells in the chamber endocardium showed ectopic F-actin labeling, and sometimes those cells also acquired cuboidal shape (Fig 8G–8G” and 8L–8L”). Taken together, these findings show that early ethanol exposure causes redistribution of Notch active cells in the heart, leading to reduced Notch activity at the AVC but ectopic activation of Notch signaling in the ventricle during AVC differentiation. Reduced Notch activity at the AVC perhaps leads to reduced F-actin positive cuboidal cells at the AVC and ectopic Notch activation in the chamber might be responsible for ectopic F-actin expression in the ventricular endocardial cells.

### Early ethanol exposure reduced Wnt activity in heart during AVC differentiation

**Fig 9:**
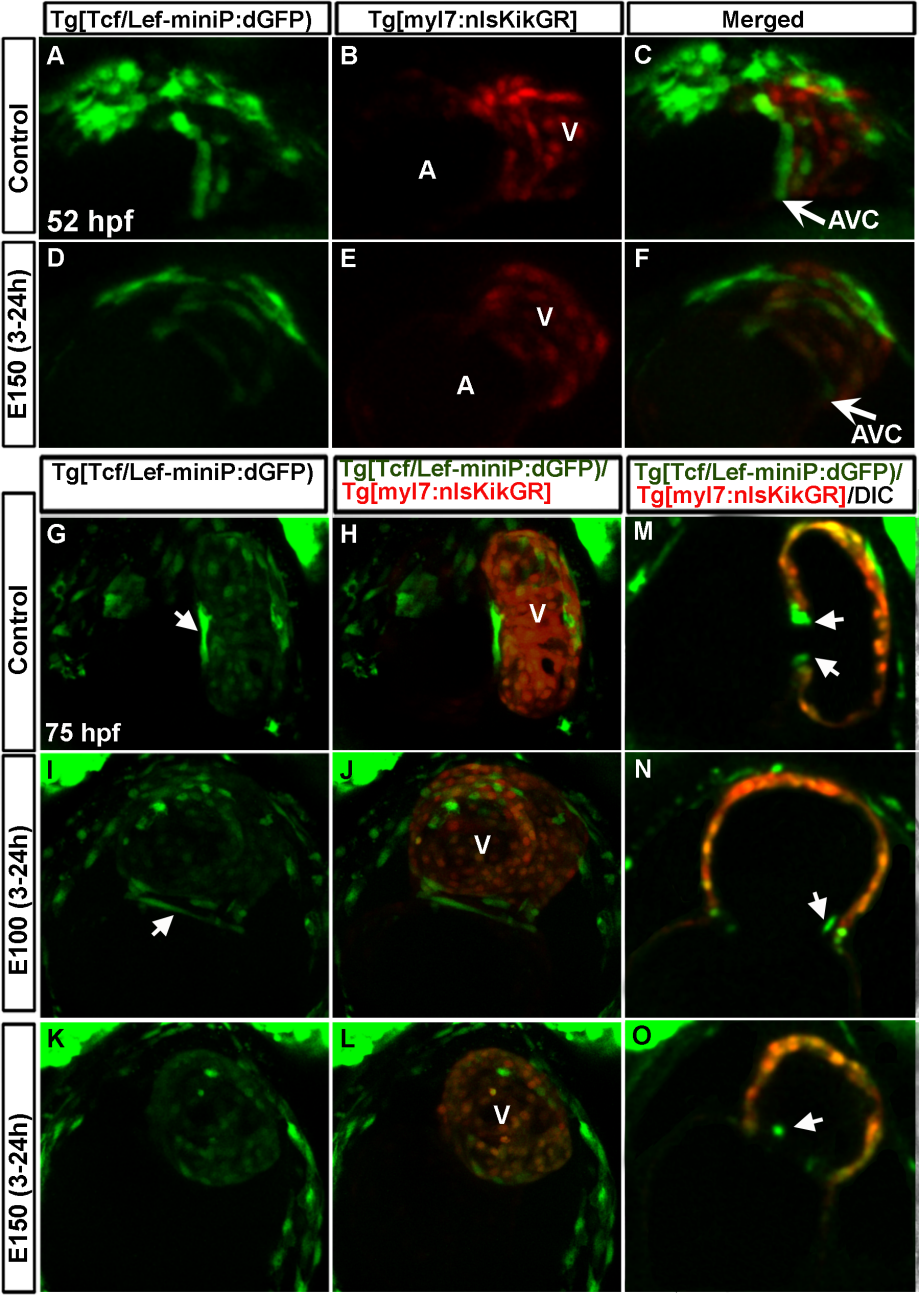
Wnt activity was reduced in heart during AVC differentiation due to embryonic ethanol exposure. (A-F) *Tg(Tcf/Lef-miniP*:*dGFP);Tg(myl7*:*nlsKikGR)* double transgenic zebrafish showed two rows of cardiomyocytes (red cells) at the AVC expressing GFP (Wnt active) in control embryos (A-C); ethanol treated embryos showed weak GFP expressing cardiomyocytes that were not restricted at the AVC (D-F) at 52 hpf. (G-L) *Tg(Tcf/Lef-miniP*:*dGFP);Tg(myl7*:*nlsKikGR)* double transgenic zebrafish showed long cardiomyocytes with strong GFP signal at the AVC and faint GFP signal in the ventricular cardiomyocytes in control embryos (G, H); ethanol exposed embryos showed weak GFP labeling at the AVC as compared to control (I-L). Arrows: Long cardiomyocytes with strong GFP signal. (M-O) Optical section of the heart of 75 hpf embryos showed GFP expression in the endocardial cells (arrowheads) at the AVC in the control embryo (M) and in ethanol treated embryos (N, O). A: Atrium, V: ventricle.

Zebrafish studies showed that spatiotemporal control of Wnt signaling during AVC differentiation is critical for AV valve formation [19]. Mouse study also identified Wnt/β-catenin signaling activity restricted to a subset of cells during endocardial cushion formation [44]. To examine Wnt activity during AVC differentiation, *Tg(Tcf/Lef-miniP*:*dGFP);Tg(myl7*:*nlsKikGR)* double transgenic zebrafish were used. *Tg(Tcf/Lef-miniP*:*dGFP)* reporter reveals transcriptional activities of four Tcf/Lef members, Lef1, Tcf7, Tcf7l1a, and Tcf7l2, controlled by Wnt/β-catenin signaling [38]. *Tg(myl7*:*nlsKikGR)* transgene labels cardiomyocytes. At 52 hpf, control embryos showed GFP expression in two rows of AVC cardiomyocytes (Fig 9A–9C). Cardiomyocytes in ethanol treated embryos showed weak GFP expression, but those cells were not restricted at the AVC (Fig 9D–9F). GFP expression was not detected in AVC endocardial cells at that stage. At 75 hpf, 2–3 elongated AVC cardiomyocytes (based on the cell count in 8 embryos) in the control embryos (towards the atrium side) showed strong GFP signal, and cardiomyocytes in the ventricle exhibited faint GFP signal (Fig 9G and 9H). Ethanol treated embryos did not show strong GFP positive cardiomyocytes at the AVC, indicating reduced Wnt activity (Fig 9I–9L). Optical sections of the heart showed GFP expression in the 2–3 endocardial cells in both the superior and inferior aspects of AVC in control embryos at 75 hpf (Fig 9M). However, ethanol treated embryos showed 0–2 GFP positive endocardial cell (based on the cell count in 8 embryos) (Fig 9N and 9O). Occasionally, GFP positive endocardial cell was not at the AVC (Fig 9O). In situ hybridization to detect Wnt target gene, *axin 2*, showed expression in the heart near the AVC in control embryos at 48 hpf. Ethanol treated embryos showed either weak expression or no expression of *axin 2* in the heart (S3A–S3C Fig). Taken together, ethanol treatment during early cardiogenesis reduces Wnt activity during valve morphogenesis.

## Discussion

Prenatal alcohol exposure produces a variety of CHDs in patients, and among those, septal defects are the most frequently seen defects [4]. Zebrafish has become a popular animal model to study FASD because of the ease of manipulation, transparency, and the conservation of genetic and regulatory pathways essential for embryogenesis [20, 24–26, 32]. There are obvious structural differences between two-chambered zebrafish and four-chambered human hearts, but zebrafish has emerged as an important model system to study cardiovascular development [5, 6]. The embryos grow outside the mother and are transparent, which facilitate the study of cardiogenesis, particularly valvulogenesis in live embryos. Zebrafish studies have dissected genetic, molecular, and cellular mechanisms of vertebrate cardiac development and function due to highly conserved cardiogenic pathway genes and cellular organizational framework [5, 6]. Zebrafish cardiac morphogenesis events are highly similar to the early stages of mammalian cardiac morphogenetic events that eventually remodel to produce a prototypic four-chambered heart by septation of the primitive two-chambered heart [5, 6]. The precursor for septation and valve formation is the endocardial cushion. Formation of endocardial cushion, the first sign of valvulogenesis, becomes evident as a swelling at the constriction of embryonic heart tube between atrium and ventricle. In zebrafish, the endocardial cells at the swelling progressively become different from the endocardial cells in the chamber lining, becoming cuboidal and expressing distinct markers, like accumulation of F-actin and Alcama marker expression [6, 14]. The characteristic cell shape change and expression of Alcama and F-actin are reliable markers for AVC endocardial cell specification and differentiation [6, 14]. This study examined the consequences of embryonic ethanol exposure on AV valves in zebrafish at different stages of life, demonstrating ethanol-induced defects in valve regulatory networks during valvulogenesis.

We demonstrated that ethanol exposure during gastrulation and somitogenesis leads to defective zebrafish AV valve morphogenesis. Ethanol exposure produces an extended AVC, as opposed to the normally compact AVC. The endocardial cushion forming cells are dispersed into the ventricle in ethanol exposed embryos, which normally reside only at the AVC. Live imaging of the embryos confirmed our histology results, ruling out the possibility of artifact due to change in valve morphology upon fixation. These ectopic AVC cells in the ventricles exhibits subsets of AVC endocardial cushion cell characteristics, like accumulation of F-actin and Alcama marker expression, but they are not always cuboidal in shape, suggesting they are different from normal differentiated endocardial cushion cells. The number of Alcama and F-actin positive cuboidal endocardial cells at the AVC, precursors of heart valve, are reduced in ethanol exposed embryos. Overall, embryonic ethanol exposure alters the properties of cells at AVC and chamber endocardium during valvulogenesis.

Our experiments showed that embryonic ethanol exposure produces persistent structural defects of AV valves in larvae and juvenile fish. Ethanol exposure produces longer and thicker AV valve leaflets in zebrafish larvae. At later stages, ethanol exposed zebrafish AV valve remodels to form four valve cusps like in control fish. However, ethanol exposure alters the position of the cusps at the AV orifice, which causes the cusps either to lie on top of one another and may not be able to form tight seal when they meet. Importantly, embryonic ethanol exposure reduces the AV valve cusp size in juvenile fish. How the longer and thicker AV valves in larvae became smaller than normal in older juvenile fish needs further study. Zebrafish AV valves grow in first two weeks, then the valve matures [45]. Ethanol exposed fish valve probably did not mature normally, as in control. WGA staining of two-months old juvenile fish also showed small and misshapen valves with wider AV orifice, suggesting that the valve defect persists at later stages of life. It is noteworthy that many severely defective embryos died at early stages, and the fish that survived until juvenile stage were probably not those most severely affected. Perhaps the defects that were observed in juvenile fish are relatively mild.

Valve development mechanisms are extremely well conserved from zebrafish to human, sharing many genes and pathways that control cardiac valve development [5, 6]. As the development proceeds to form atrium and ventricular chambers, initial heart tube subdivided into four segments, two chambers, AVC region and the OFT [5, 6]. Bmp and Notch signaling are required for initial patterning of AVC and to define the AVC and chamber regions [15]. Luna-Zurita et al. showed that myocardial Bmp and endocardial Notch signal integration establish the valve forming field between two chamber domains [46] and generate AVC unique myocardium capable of producing epithelial-to-mesenchymal transition (EMT)-inducing ligands and responsive endocardium [16, 46–49]. AVC differentiation depends on the cross-talk between myocardial and endocardial cells at the AVC, involving multiple signaling pathways including Bmp, Notch, and TGF-ß signaling [50–55]. In mice BMP2 and BMP4 are expressed in developing AVC [10]. In zebrafish, *bmp4* expression is high at the AVC but the chamber cardiomyocytes do not express *bmp4* during valve formation. *Notch1* is initially expressed in cardiac crescent and throughout endocardium in mouse, but later the expression is elevated at AVC and OFT [56]. *Notch2* is also expressed at AVC [52]. Notch target genes *Hey1* and *Hey2* are expressed in the AVC endocardium and chamber myocardium [52]. In zebrafish, *notch1b* is initially expressed in all ventricular endocardial cells, but later becomes restricted at the AVC endocardium [6, 14, 16]. Myocardial-derived Bmp initiates local EMT at AVC [47]. Bmp signaling induces *Tbx2* (Bmp signaling target) expression and the expression of TGF-ß pathway components at the AVC [57]. Deletion of *Bmp2* reduces the expression of *Notch1* and *Snail* expression in the AVC endocardium, suggesting that Bmp signaling maintains Notch signaling during cardiac cushion formation [47]. Notch signaling also regulates Bmp signaling and influences TGF- ß pathway components during valve formation. Notch target genes *Hey1* and *Hey2* suppress *Bmp2* and *Tbx2* expression from the chambers and restricts their expression at the AVC [50, 52, 54]. Over all, complex inter-relationship between these signaling pathways promotes EMT and proper formation of AV valves.

Our experiments showed that embryonic ethanol exposure prevents regionalization of Bmp activity at the AVC during valvulogenesis. Experiments detecting Bmp ligand and measuring Bmp activity in the heart during endocardial cushion formation showed similar results, validating our findings. Ethanol causes chamber myocardial and endocardial cells to achieve some characteristics of valvular cells. It was shown that Bmp regulates the production of cushion extracellular matrix by activating *has2* [17, 57]. High Bmp activity in the chamber cells in ethanol treated embryos during endocardial cushion formation presumably leads to secretion of more extracellular matrix that leads to thicker cardiac jelly in the extracellular space between myocardium and endocardium. Notably, Bmp activity remains low in ethanol exposed embryos during heart tube morphogenesis stages. Transgenic embryos reporting Bmp activity showed reduced GFP signal in heart tube until the onset of endocardial cushion formation.

Similar to Bmp, we found that Notch activity is not restricted at AVC in endocardial cells. Embryonic ethanol exposure changes the distribution of high and low Notch active cells in the endocardium. Instead of forming clusters at the AVC, high Notch active cells were dispersed throughout the ventricle. Frequently, these ectopic Notch active cells were in the posterior region of the ventricle. Previous murine study showed that constitutive Notch1 activation in endocardium promotes ectopic noninvasive EMT and confer valvular features to otherwise non-vavlular ventricular endocardium [46]. Our experiments using *Tg(TP1*:*mCherry)* Notch reporter line showed similar results. Highly active Notch cells arranged in a row at the AVC in control embryos expressed high level of F-actin and were cuboidal in shape, suggesting those cells were valve forming differentiated endocardial cushion cells. But ethanol exposure reduced the number of highly active Notch cells at the AVC and subsequently reduced F-actin positive cuboidal cells at the AVC. On the other hand, chamber cells with high Notch activity exhibited F-actin staining, suggesting those cells acquired subset of valvular characteristics.

In addition to their roles in AV valvulogenesis, Bmp and Notch signaling play crucial roles throughout cardiogenesis. Bmp signaling is required for cardiac specification, differentiation, endocardial differentiation, cardiac looping, chamber morphogenesis, and OFT septation during embryogenesis [5, 6, 58–62]. Notch1 signaling is required for the progression of differentiation of the endocardium, cardiac looping, and OFT development [16, 63, 64]. Our previous study showed that embryonic ethanol exposure disrupts cardiac specification, reduces cardiac looping, and produces dysmorphic heart chambers [32]. Ethanol exposure also reduces endocardial differentiation and produces smaller, defective OFT (Sarmah et al., unpublished data). We hypothesize that Bmp and Notch signaling are major signaling pathways disrupted by ethanol exposure during cardiogenesis. Further analyses manipulating these signaling pathways by genetic method or pharmacological agents in ethanol treated embryos will provide insight and help us pinpoint the origin of defects.

Recent study by Borsoda et al. showed that Wnt/β-catenin signaling is not required for EMT at AVC in mice, but it promotes AVC cushion expansion following EMT [65]. A zebrafish study also showed that Wnt/β-catenin signaling is operative in the valve forming myocardial and endocardial cells. Constitutive activation of Wnt/β-catenin signaling is associated with proliferation of endocardial cushion cells [19]. Our transgenic Wnt-reporter line experiments showed prominent Wnt/β-catenin activity in the AVC cardiomyocytes and in the endocardial cushion cells at later stages of valve development, after Bmp and Notch showed restricted activity at the AVC. We showed that ethanol exposure reduced Wnt/β-catenin activity during valve development. Wnt activity was reduced by ethanol exposure during cardiac speciation in avian embryos [34]. The expression of canonical Wnt-modulated genes *Hex1* and *Islet1* were suppressed in ethanol-exposed quail embryos during cardiac cell specification [34].

Our results show that embryonic ethanol exposure alters Bmp, Notch and Wnt/β-catenin signaling networks during heart valve development. The genesis of these altered regulatory networks by embryonic ethanol exposure is still unknown. We speculate that ethanol exposure alters the incorporation of second heart field and neural crest derived cells, which alters the cellular environment in the heart. Altered cardiomyocyte populations might change signaling in the remaining cardiomyocytes. Those cardiomyocytes fail to suppress Bmp activity within the ventricular chamber, expanding valvular territory. These altered cardiomyocytes influence underlying endocardial cells leading to defective valvulogenesis. Alternatively, ethanol exposure could directly affect the endocardial cells, producing an altered phenotype during valvulogenesis. Additional studies are needed to understand the primary causes for all these defects. Nevertheless, our study systematically followed the consequences of embryonic ethanol exposure on AV valve development, linking those defects to faulty valvulogenesis regulatory pathways.

## Supporting Information

S1 Fig Phospho-Smad-1/5/9 positive cardiomyocytes were seen throughout the ventricle in ethanol-exposed embryos. (A-I) 3D reconstruction of confocal sections of phospho-Smad-1/5/9 (A, D, G) and MF20 (B, E, H) double immunostained embryos showed Bmp responsive phospho-Smad-1/5/9 positive cardiomyocytes at the base of the atrium and in the inner curvature of the ventricle in control embryos (A); regionalization of phospho-Smad-1/5/9 positive cardiomyocytes were not evident in ethanol exposed embryos (D, G); A’, D’, G’: magnified images of boxed areas of A, D, G.
(TIF)

S2 Fig Expression of *her2* in the heart. (A-C) *her2* expression in the control heart (A); *her2* expression in ethanol treated embryo hearts (B, C). Arrow: heart.
(TIF)

S3 Fig Expression of *axin2* in the heart. (A-C) In situ hybridization detecting *axin 2* showed expression of the gene in the heart near AVC in control embryos (A); weak expression in E100 ethanol treated embryo (B) and no expression in E150 ethanol treated embryo. Arrows: pointing *axin 2* expression in the heart.
(TIF)

S1 Movie Beating heart of 3 dpf control zebrafish embryo. SPIM movie of the beating heart of a control *Tg(fli1*:*EGFP)* embryo taken at 100 fps and shown at 16 fps. Orientation of the embryo: Ventricle is facing upward.
(MOV)

S2 Movie Beating heart of 3 dpf ethanol treated (E100) zebrafish embryo. SPIM movie of the beating heart of an ethanol treated (E100) *Tg(fli1*:*EGFP)* embryo taken at 100 fps and shown at 16 fps. Orientation of the embryo: Ventricle is facing upward.
(MOV)

S3 Movie Beating heart of 3 dpf ethanol treated (E150) zebrafish embryo. SPIM movie of the beating heart of an ethanol treated (E150) *Tg(fli1*:*EGFP)* embryo taken at 100 fps and shown at 16 fps. Orientation of the embryo: Ventricle is facing upward.
(MOV)
