# Supplementary figures and images for: Embryonic Ethanol Exposure Dysregulates BMP and Notch Signaling, Leading to Persistent Atrio-Ventricular Valve Defects in Zebrafish
Source: PLoS One. 2016 Aug 24;11(8):e0161205. doi: 10.1371/journal.pone.0161205 (PMC4996461; doi:10.1371/journal.pone.0161205)

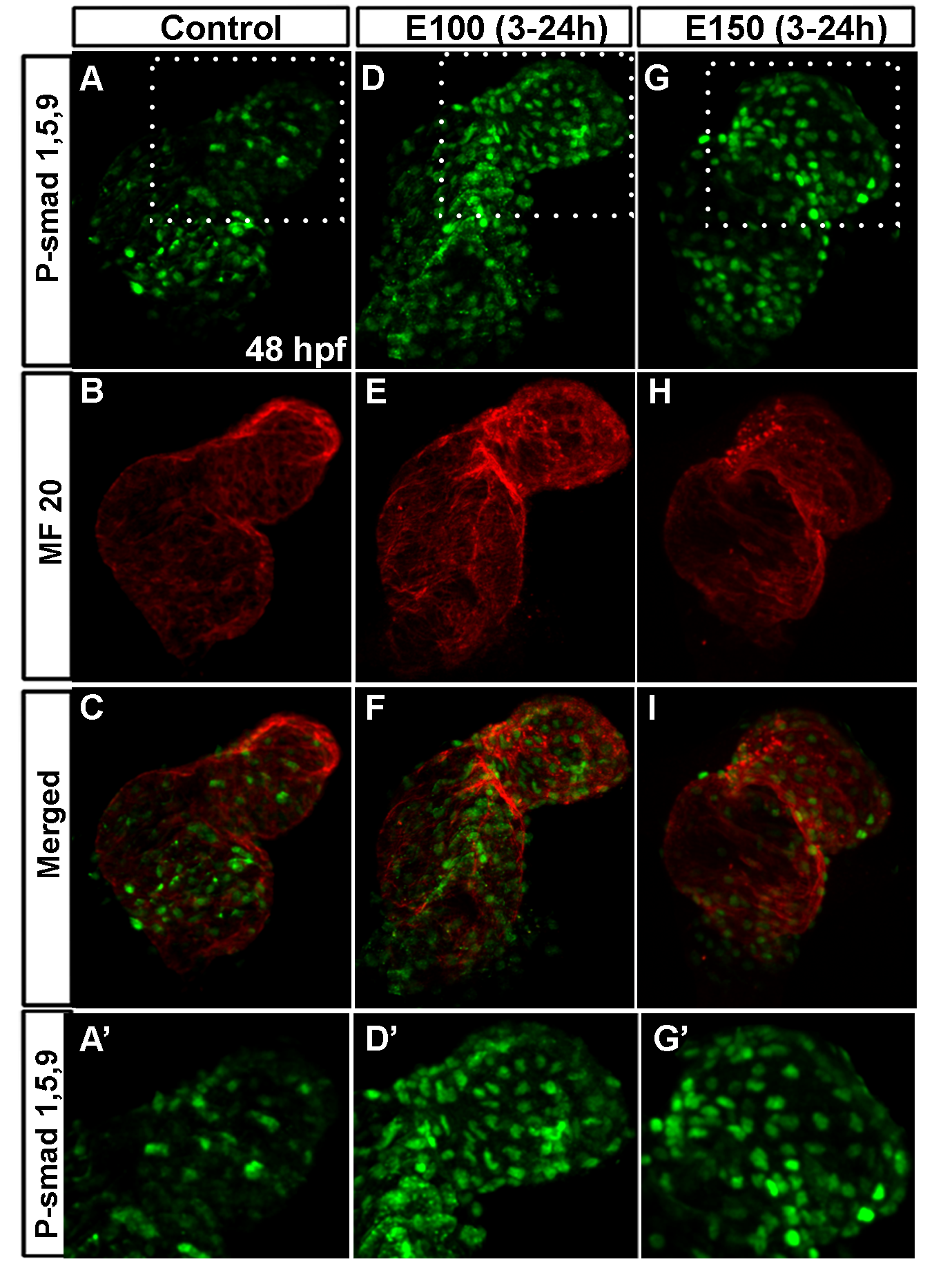

Supplement: S1 Fig — (A-I) 3D reconstruction of confocal sections of phospho-Smad-1/5/9 (A, D, G) and MF20 (B, E, H) double immunostained embryos showed Bmp responsive phospho-Smad-1/5/9 positive cardiomyocytes at the base of the atrium and in the inner curvature of the ventricle in control embryos (A); regionalization of phospho-Smad-1/5/9 positive cardiomyocytes were not evident in ethanol exposed embryos (D, G); A’, D’, G’: magnified images of boxed areas of A, D, G. (TIF) [file pone.0161205.s001.tif]

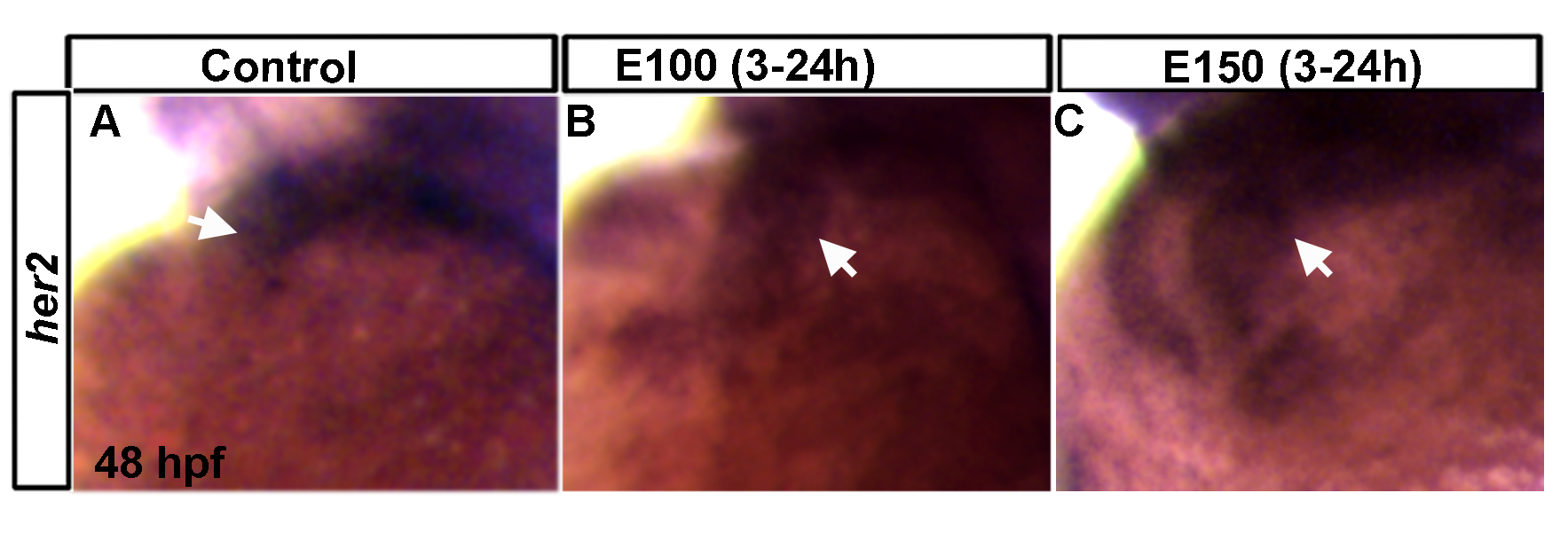

Supplement: S2 Fig — (A-C) her2 expression in the control heart (A); her2 expression in ethanol treated embryo hearts (B, C). Arrow: heart. (TIF) [file pone.0161205.s002.tif]

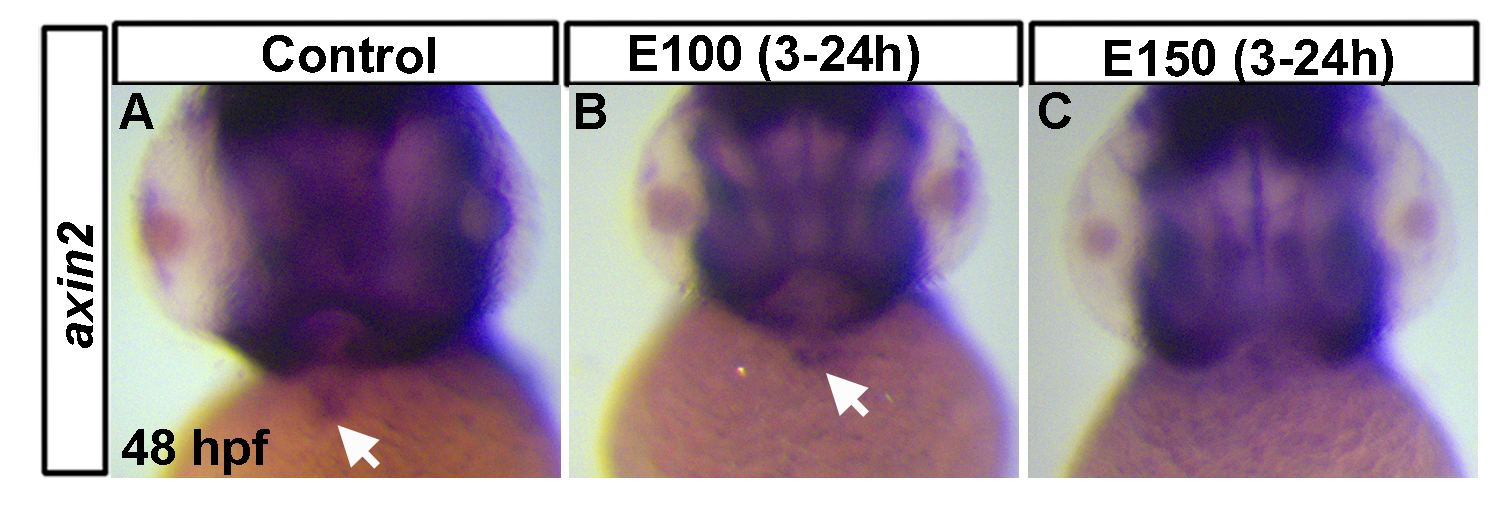

Supplement: S3 Fig — (A-C) In situ hybridization detecting axin 2 showed expression of the gene in the heart near AVC in control embryos (A); weak expression in E100 ethanol treated embryo (B) and no expression in E150 ethanol treated embryo. Arrows: pointing axin 2 expression in the heart. (TIF) [file pone.0161205.s003.tif]
